# Rehydration characteristics of dehydrated West African pepper (*Piper guineense*) leaves

**DOI:** 10.1002/fsn3.149

**Published:** 2014-07-22

**Authors:** Laura C Okpala, Constance A Ekechi

**Affiliations:** Department of Food Science and Technology, Ebonyi State UniversityAbakaliki, Ebonyi State, Nigeria

**Keywords:** Blanching, drying, Peleg's constant, rehydration

## Abstract

The rehydration characteristics of dehydrated West African pepper leaves were investigated at hydration temperatures of 28, 60, 70, and 80°C. Four treatments were given to the leaves: blanched and sun dried, unblanched and sun dried, blanched and shade dried, and unblanched and shade dried. The hydration process of the dehydrated leaves was adequately described by the Peleg's equation. As the hydration temperature increased from 28 to 70°C, there was a significant decrease in the Peleg's constant *K*_1_, while for most of the leaves the Peleg's constant *K*_2_ varied with temperature. Rehydration ratio values ranged from 3.75 in blanched shade dried leaves to 4.26 in unblanched sun dried leaves with the unblanched leaves generally exhibiting higher ratios than the blanched leaves.

## Introduction

West African pepper also known as Guinea pepper or Ashanti pepper is a herbaceous climber belonging to the Piperaceae family. It is a perennial plant that is characterized by heart-shaped leaves. Its leaves are used as flavoring for stews. Owing to their short shelf life and seasonal nature, the leaves are often dried to preserve them. Such leaves, however, must be rehydrated prior to its use. Rehydration is a process which is aimed at restoring the properties of a raw material when the dried material comes in contact with water (An et al. 2013). Rehydration of food materials is often carried out by soaking the dried material in water (Garcia-Pascual et al. 2005). Rehydration may be regarded as a measure of the injury to the material which occurs as a result of drying and treatment that precedes dehydration (McMinn and Magee 1997). The extent of rehydration depends on the degree of structural and cellular disruption (Krokida and Marinos-Kouris 2003). Jayaraman et al. (1990) noticed irreversible cellular rupture and dislocation, which led to the loss of integrity and consequently, a dense structure of collapsed, greatly shrunken capillaries with reduced hydrophilic properties, as seen by the inability to imbibe enough water to fully rehydrate. The rehydration characteristics of dried food materials are used as a quality parameter and show whether physical and chemical changes occurred during the drying process due to process conditions, pretreatments and sample composition (Lewicki 1998). Physical and chemical changes that occur during drying affect the quality of the dried material such that even with the addition of water, the properties of the raw material cannot be restored (Krokida and Marolis 2001).

Studying the rehydration characteristics of food materials is, therefore, important, as this information is necessary to optimize processes from a quality viewpoint since rehydration is a key quality aspect for those dried products that have to be reconstituted before their consumption (Garcia-Pascual et al. 2006). In an attempt to simplify the mode of water absorption by food materials, a nonexponential empirical formula was proposed and became known as the Peleg's equation (Peleg 1998).

Peleg's equation can be written thus:


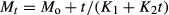
(1)

As *t* → ∞


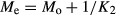
(2)

Linearizing equation (1) will give:


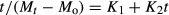
(3)

where *M*_*t*_ is moisture content at time *t* (% db), *M*_o_ is initial moisture content (% db), *t* is rehydration time (min), *K*_*1*_ is the Peleg's rate constant (min/%mc db), *K*_*2*_ is the Peleg's capacity constant (%mc db)^−1^, and *M*_e_ is the equilibrium moisture content (% db). For the equation fitting, the curvilinear portion of the hydration data is often employed. This is because the Peleg's equation is applicable to the curvilinear segment of the sorption curve (Maharaj and Sankat 2000).

The applicability of Peleg's equation has been demonstrated for some leafy vegetables. Maharaj and Sankat (2000) reported that Peleg's equation could adequately describe the water absorption characteristics of both blanched and unblanched dasheen leaves in the temperature range of 60 and 100°C.

The objective of this study was to examine whether Peleg's equation can be used in modeling the water absorption behavior of blanched and unblanched West African pepper leaves dried under different conditions. The rehydration abilities of the reconstituted leaves were also investigated.

## Materials and Methods

Freshly harvested West African pepper leaves were washed, destalked, and sliced using a sharp kitchen knife to sizes ranging between 15 and 20 mm. The leaves were divided into four portions: two portions were blanched in water at 100°C for 10 sec while the other two portions were not blanched. A portion each from the blanched and unblanched samples were sun dried while the other two portions were dried under a well-ventilated shaded area. The samples were thereafter referred to as blanched sun dried, unblanched sun dried, blanched shade dried, and unblanched shade dried.

The initial moisture content of the leaves was determined by drying 5 g of the dried leaves in an air oven at 100°C until a constant weight was obtained.

An initial amount of 1 g of dehydrated leaves was used for each experiment. The leaves were rehydrated by immersion in 250 mL beakers filled with water. Four rehydration temperatures were used: 28, 60, 70, and 80°C (±1°C). Temperature was maintained by placing beakers in a thermostatically controlled water bath. Beakers were withdrawn from the water bath after 15, 30, 45 min, 1 h, and thereafter every 30 min. After specified soaking times, the hydrated leaves were blotted free of excess surface moisture with an absorbent cloth and weighed. The increase in the weight was taken as the amount of water absorbed. The linearized form of Peleg's equation was used to fit the experimental data within the curvilinear segments of graphs obtained and away from equilibrium conditions (i.e., during the period of the increase in moisture content) as described by Maharaj and Sankat (2000). Rehydration of leaves continued until the difference between two consecutive weighings was insignificant. All the samples were studied in duplicate and percent moisture was recorded on dry basis (%db). Rehydration ratio of the different leaves was determined using the method described by Ranganna (1986).


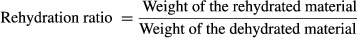


## Results and Discussion

**Figure 1:**
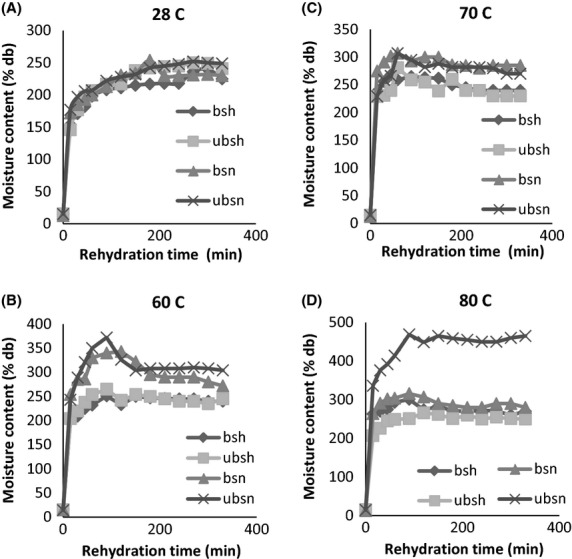
Water absorption characteristics of blanched and unblanched West African pepper leaves dried under different conditions at different temperatures. bsh = blanched and shade dried; ubsh = unblanched and shade dried; bsn = blanched and sun dried; ubsn = unblanched and sun dried.

The rehydration curves of the blanched and unblanched leaves dried under the sun and shade are shown in Figure1A–D. It was observed that the initial rate of water uptake increased as the temperature increased. This suggests that rapid rehydration can be achieved when the temperature of the water is high. Figure1A–D shows that a prolonged soaking time does not contribute to further water uptake. Maharaj and Sankat (2000) made a similar observation during the rehydration of dehydrated dasheen leaves. At all the temperatures used in the study, it was observed that the unblanched sun dried leaves had the highest uptake of water. A summary of the linear regression models fitted to the data at the different hydration temperatures is shown in Table1. The coefficients of determination were found to be high in all cases (*R*^2^ > 0.97) indicating a good fit of the experimental data to Peleg's model at all the examined temperatures.

**Table 1:** Summary of linear regression models fitted *t*/(*M*_*t*_ − *M*_o_) versus *t* for West African pepper leaves hydrated at 28–80°C.

Treatment	Temperature (°C)	Estimated slope *K*_2_ (% mc db)^−1^	*P*	*R*^2^
Blanched sun dried	28	4.17 × 10^−3^	0.000	0.989
	60	3.00 × 10^−3^	0.000	0.993
	70	3.33 × 10^−3^	0.000	0.995
	80	3.31 × 10^−3^	0.000	0.998
Unblanched sun dried	28	4.09 × 10^−3^	0.000	0.988
	60	2.70 × 10^−3^	0.000	0.978
	70	3.40 × 10^−3^	0.000	0.973
	80	2.20 × 10^−3^	0.000	0.985
Blanched shade dried	28	4.47 × 10^−3^	0.000	0.993
	60	4.20 × 10^−3^	0.000	0.990
	70	3.97 × 10^−3^	0.000	0.998
	80	3.49 × 10^−3^	0.000	0.997
Unblanched shade dried	28	3.81 × 10^−3^	0.000	0.986
	60	3.89 × 10^−3^	0.000	0.987
	70	3.99 × 10^−3^	0.000	0.992
	80	3.97 × 10^−3^	0.000	0.997

### Peleg's constant *K*_2_

Values obtained for the Peleg's constant *K*_2_ are presented in Table1. Peleg's constant *K*_2_ is related to maximum water absorption capacity, that is, the lower the *K*_2_, the higher the water absorption capacity (Turhan et al. 2002). Abu-Ghannam and McKenna (1997) reported that *K*_2_ is a constant that defines the equilibrium moisture content. In this study, the relationship between temperature and *K*_2_ was insignificant (*P *> 0.05) and the values of *K*_2_ were not always constant with temperature. The blanched sun dried, unblanched sun dried, and blanched shade dried leaves had highest *K*_2_ values at 28°C. This means that the maximum water absorption capacity of these leaves was lowest at 28°C. However, for the unblanched shade dried leaves, the Peleg's constant *K*_2_ was fairly constant at all the temperatures. Sopade et al. (1994) reported that *K*_2_ values for some cowpea varieties were constant with soaking temperature; while for dasheen leaves, it was observed that *K*_2_ changed with temperature for steam blanched and alkali blanched leaves (Maharaj and Sankat 2000).

Peleg's *K*_2_ values were used to calculate the equilibrium moisture content of the leaves according to equation (2) (Table2). It was observed that at almost all the temperatures, the sun dried leaves had higher values than their shade dried counterparts regardless of whether they were blanched or not. Equilibrium moisture content of all the hydrated leaves was lower than those reported for dasheen leaves (Maharaj and Sankat 2000). There was a close agreement in the equilibrium moisture content values for blanched sun dried, unblanched sun dried, blanched shade dried, and unblanched shade dried leaves determined by experimental and Peleg's equation at hydration temperatures of 28, 60, 70, and 80°C.

**Table 2:** Equilibrium moisture content of hydrated West African pepper leaves determined experimentally and by predictive methods.

Treatment	Temperature (°C)	Observed equilibrium moisture content (% db)	Predicted equilibrium moisture content (% db)
Blanched sun dried	28	233	252
	60	340	348
	70	300	313
	80	305	315
Unblanched sun dried	28	250	260
	60	320	385
	70	285	309
	80	465	467
Blanched shade dried	28	235	234
	60	250	253
	70	263	267
	80	275	302
Unblanched shade dried	28	245	278
	60	250	273
	70	257	266
	80	260	267

### Peleg's rate constant *K*_1_

The Peleg's constant *K*_1_ decreased significantly as the hydration temperature increased from 28 to 70°C (Table3). This suggests a corresponding increase in the initial water absorption rate (Turhan et al. 2002). The reciprocal of *K*_1_ is equivalent to the initial rate of hydration (Maharaj and Sankat 2000). It was observed that the values of 1/*K*_1_ were significantly (*P < 0*.05) higher at 70 than at 80°C for the blanched and unblanched shade dried leaves while values for the blanched and unblanched sundried leaves were not significantly (*P > 0*.05) different at 70 and 80°C. The lower values of 1/*K*_1_ at 80°C may probably be due to the fact that the high soaking water temperature resulted in shrinkage and/or loss of elastic properties of cell tissues rather than causing an opening up of pores, which invariably reduced the rate of initial hydration (Tunde-Akintunde 2008).

**Table 3:** Peleg's *K*_1_ values for West African pepper leaves hydrated at 28–80°C.

Treatment	Temperature (°C)	*K*_1_	1/*K*_1_
Blanched sun dried	28	4.21 × 10^−2^	24
	60	1.45 × 10^−2^	69
	70	6.00 × 10^−3^	167
	80	7.00 × 10^−3^	143
Unblanched sun dried	28	4.53 × 10^−2^	22
	60	2.01 × 10^−2^	50
	70	1.2 × 10^−2^	83
	80	1.2 × 10^−2^	83
Blanched shade dried	28	5.73 × 10^−2^	17
	60	1.70 × 10^−2^	59
	70	6.1 × 10^−3^	164
	80	1.06 × 10^−2^	94
Unblanched shade dried	28	8.07 × 10^−2^	12
	60	1.96 × 10^−2^	51
	70	8.70 × 10^−3^	115
	80	1.60 × 10^−2^	63

Various researchers have shown that *K*_1_ is a temperature-dependent constant (Sopade et al. 1994; Maharaj and Sankat 2000). In this study, for most of the leaves, a significant linear relationship (*R*^2^ ranged between 0.91 and 0.96; *P *< 0.05) was found to exist between temperature and *K*_1_ which agrees with the findings of previous studies. However, for the unblanched shade dried leaves, *K*_1_ was not found to be temperature dependent (*R*^2^ = 0.88; *P* = 0.06). Abu-Ghannam and McKenna (1997) observed that *K*_1_ for unblanched beans was not temperature dependent as this was reflected in a low correlation coefficient (*R*^2^ = 0.61). This suggests that *K*_1_ could also be dependent on other properties of the food materials in question. Initial hydration rates as determined by 1/*K*_1_ were highest for blanched sun dried leaves at all the temperatures studied. The blanched shade dried leaves also exhibited higher initial hydration rates than the unblanched shade dried leaves. This may suggest that blanching can improve initial hydration rates, and therefore the rehydration characteristics of West African pepper leaves.

### Rehydration ratio

Rehydration is one way to analyze dried products. A high value of rehydration ratio means the dried product has a good quality because the pores allow water to reenter the cells (Noomhorm 2007). Rehydration ratio ranged from 3.75 in blanched shade dried leaves to 4.26 in unblanched sun dried leaves (Table4). Generally, it was observed that the unblanched leaves had higher ratios than the blanched leaves. Rajeswari et al. (2011) observed a similar trend with amaranthus leaves. It was also observed that sun drying led to higher rehydration ratios than shade drying.

**Table 4:** Rehydration ratio of hydrated West African pepper leaves.

Treatment	Rehydration ratio
Blanched sun dried	3.77
Unblanched sun dried	4.26
Blanched shade dried	3.75
Unblanched shade dried	3.83

Values are means of duplicate determinations.

## Conclusion

The Peleg's model could adequately describe the water absorption of blanched sun dried, unblanched sun dried, blanched shade dried, and unblanched shade dried leaves between 28 and 80°C. The model could also predict the equilibrium moisture content of the leaves. The Peleg's constant *K*_1_ decreased significantly as the hydration temperature increased from 28 to 70°C. The Peleg's constant *K*_2_ was not constant with temperature for most of the leaves. Only the unblanched shade dried leaves, exhibited fairly constant values at all the temperatures.

## Conflict of Interest

None declared.
